# Efficacy of a polyvalent immersion vaccine against *Flavobacterium psychrophilum* and evaluation of immune response to vaccination in rainbow trout fry (*Onchorynchus mykiss* L.)

**DOI:** 10.1186/s13567-017-0448-z

**Published:** 2017-08-18

**Authors:** R. Hoare, T. P. H. Ngo, K. L. Bartie, A. Adams

**Affiliations:** Institute of Aquaculture, University of Stirling, Stirling, FK9 4LA UK

## Abstract

Rainbow trout fry syndrome (RTFS) is a disease caused by the Gram-negative bacterium *Flavobacterium psychrophilum,* responsible for significant economic losses in salmonid aquaculture worldwide. The diversity of *F. psychrophilum* isolates and the inherent difficulties in vaccinating juvenile fish has hampered the development of a vaccine for RTFS. Disease episodes tend to occur between 10–14 °C with necrotic lesions often seen on the skin surrounding the dorsal fin and tail. At present no commercial vaccines are available for RTFS in the UK, leaving antibiotics as the only course of action to control disease outbreaks. The current work was performed as a pilot study to assess the efficacy of a polyvalent, whole cell vaccine containing formalin-inactivated *F. psychrophilum*, to induce protective immunity in rainbow trout fry. Duplicate groups of 30 trout (5 g) were immersed in 1 L of the vaccine for 30 s. Samples were taken 4 h, day 2 and 7 post-vaccination (pv) of skin mucus, tissues for histology and gene expression analysis; serum and histology samples were taken 6 weeks pv. A booster vaccination was given at 315 degree days (dd) also by immersion. Challenge was by immersion with a heterologous isolate of *F. psychrophilum* 630 dd post primary vaccination. The vaccine provided significant protection to the trout fry with a RPS of 84% (*p* < 0.0001). Detection of increased numbers of IgT positive cells in systemic organs, up-regulation of *IgT* expression in hind-gut and an increase in total IgT in serum was observed in vaccinated fish; however a functional role of IgT in the observed protection remains to be demonstrated.

**Electronic supplementary material:**

The online version of this article (doi:10.1186/s13567-017-0448-z) contains supplementary material, which is available to authorized users.

## Introduction

Rainbow trout fry syndrome (RTFS) caused by *Flavobacterium psychrophilum* is one of the most significant disease problems facing the aquaculture industry worldwide, with infections of salmonids reported in Europe, North America, Australia, Chile, Peru, Japan, Korea [1]. *Flavobacterium psychrophilum* is a highly heterogeneous pathogen and a limited number of commercial vaccines are currently available (Chile, Norway) to counteract this devastating disease which are not suitable for juvenile fish [2]. The only course of action if an episode occurs in fry is antibiotic treatment which has led to increased levels of antibiotic resistance [3–5], highlighting the urgent need for prophylactic treatments against RTFS suitable for administration to juvenile fish and providing cross-protection. As RTFS affects salmonid fry when they are too small to vaccinate by injection, oral and immersion vaccination allows the only option for mass delivery of a vaccine to provide protection for the fish as early as possible. Recently, the expression of immunoglobulin isotypes in response to immersion vaccination of rainbow trout weighing 35 g to live attenuated *F. psychrophilum* was investigated [6], while studies on vaccinated fry, especially using inactivated whole cell vaccines, are lacking. Existing immersion and oral vaccines for *Yersinia ruckeri* and *Vibrio anguillarum* have demonstrated the minimum size of the onset of adaptive immunity in salmonids tends to be between 0.5 and 2.5 g [7] suggesting that it should be possible to stimulate immunity in salmonid fry against *F. psychrophilum* by these vaccination methods.

An initial study was carried out to determine the heterogeneity of *F. psychrophilum* isolates circulating in farms predominantly in the UK. This was achieved by genotyping and serotyping a large collection of isolates (293 UK, 16 Europe, 4 USA, 2 Chile) collected from rainbow trout (*Onchorynhcus mykiss*), Atlantic salmon (*Salmo salar*) and coho salmon (*Onchorynchus kisutch*) and the results are described elsewhere [8]. Large strain variation was found among the isolates studied and these data were used to choose three representative strains from the collection (two from trout and one from salmon; three serotypes, three pulsotypes) for inclusion in a vaccine which could provide cross-protection against RTFS.

The lack of an effective and reproducible immersion challenge model has also hampered the development of an immersion vaccine for *F. psychrophilum* [9, 10]. A standardised challenge model exists via the intramuscular route [11] however this is not an appropriate method to test the efficacy of immersion vaccines as this route bypasses the immunity stimulated via mucosal vaccination. Previous challenge models using scarification [9, 10, 12, 13] or stress have had some success but with low mortalities. A recent study incorporating pre-treatment of trout fry with hydrogen peroxide in the challenge model resulted in mortalities of up to 30% [14]. In the current study our aim was to improve the challenge model described by [14], to conduct a pilot study to assess the efficacy of our polyvalent immersion vaccine and to investigate the immune response following vaccination. To our knowledge this is the first report of the successful immersion vaccination of rainbow trout fry against RTFS tested by immersion challenge using a heterologous strain of *F. psychrophilum*.

## Materials and methods

### Fry

Trout fry (2 g) were obtained from a commercial fish farm with a spring-fed hatchery in Scotland and transported to the aquarium at the Institute of Aquaculture, Stirling. The fish were allowed to acclimatise for 8 weeks in a 300 L flow-through (5 L min^−1^) de-chlorinated tap water at 15 °C before vaccination. The fish were fed 2% body weight/day (Inicio feed, 1.1 mm, BioMar). The *F. psychrophilum*-free status of the fry was determined by streaking head kidney and spleen samples of ten fish on to Modified Veggietone (MV) medium [veggitones GMO-free soya peptone (Oxoid, UK), 5 g L^−1^; yeast extract (Oxoid, UK), 0.5 g L^−1^; magnesium sulphate heptahydrate (Fisher chemicals, UK), 0.5 g L^−1^; anhydrous calcium chloride (BHD), 0.2 g L^−1^; dextrose (Oxoid, UK), 2 g L^−1^; agar (solid medium; Oxoid, UK), 15 g L^−1^; pH 7.3] at 15 °C for 72–96 h. The presence of *F. psychrophilum* was examined using a nested PCR method targeting the 16S ribosomal RNA gene, as described by [8, 15]. All experimental procedures with live fish were carried out in accordance with the UK Animals (Scientific Procedures) Act, 1986 and associated guidelines, EU Directive 2010/63/EU for animal experiments and were approved by the Ethics Committee of the Institute of Aquaculture, University of Stirling, UK.

### Characterisation and selection of *F. psychrophilum* isolates for inclusion in the vaccine

A total of 293 *F. psychrophilum* isolates, collected from 27 sites within the UK between 2005 and 2015, were characterised using four genotyping methods and a serotyping scheme as reported previously [8]. The three isolates included in the vaccine originated from three diverse sites within the UK, within two fish hosts of rainbow trout and Atlantic salmon, belonged to three unique pulsotypes, represented the three serotypes found (Fp^T^, Fd and Th), two (GTG)_5_-PCR types and the prevalent plasmid profile p1 and 16S rRNA 259-93 CSF allele [8].

### Preparation of formalin inactivated bacteria

Two strains of *F. psychrophilum* recovered from trout and one recovered from Atlantic salmon in the UK during 2013 were used to make the whole cell vaccine (AVU-1T/13, serotype Fd; AVU-2T/13, serotype Th; and AVU-3S/13, serotype Fp^T^). A single colony of each strain was inoculated into MV broth (2 mL) at 15 °C for 72 h. This culture was used to inoculate the bulk culture of 200 mL per strain. The three cultures were individually pelleted by centrifugation at 3000 × *g*, 15 min, washed with sterile PBS, re-suspended in 50 mL of PBS and formalin-inactivated (0.5% formaldehyde) for 72 h, 4 °C with gentle stirring. Formalin was inactivated by addition of 15% sodium metabisulphite to each culture (1/100), 4 °C for 72 h with gentle stirring. The cultures were pelleted and washed twice (as above) and the OD^525^ adjusted to 1.0. The three cultures were mixed in equal parts (33.33 mL) to form the whole cell vaccine at a final concentration of 1 × 10^9^ colony forming units (CFU) mL^−1^. The purity of each culture and confirmation of inactivation was monitored by streaking bacterial samples onto MV agar plates, 15 °C for 72–96 h.

### Immersion vaccination and sampling

The formalin-inactivated vaccine was diluted 1:10 to give 1 L of vaccine at 1 × 10^8^ CFU mL^−1^. Duplicate groups of 30 fish (4.72 ± 1 g) were immersed in the diluted vaccine for 30 s before being transferred into 25 L flow-through tanks with aeration, 15 °C. Control groups were immersed in 1 L of tank water for 30 s. Prior to sampling fish were starved for 24 h and euthanised by an over dose of benzocaine (Sigma). Fish were sampled (n = 6) for skin mucus and tissues at 4 h post-vaccination (pv), 2 and 7 days pv. Skin mucus was sampled by placing three fish from each duplicate tank into a plastic bag containing 5 mL of Tris-buffered saline (10 mM Tris base, 0.5 M NaCl pH 7.5) and gently massaging for 2 min. Fish were removed and mucus was collected into a centrifuge tube and placed on ice while sampling. Any mucus samples contaminated with blood were discarded. Mucus was centrifuged at 4000 × *g* for 15 min, aliquoted into sterile tubes and stored at −70 °C. Tissues (gill, skin, head-kidney, spleen, hind-gut) from three fish from each duplicate group (n = 6) were sampled at 4 h pv, 2 and 7 days pv for gene expression and histology (n = 3). Tissue samples were collected and immediately fixed in formaldehyde in PBS (100 mL of 35% formaldehyde and 900 mL of DW) for histology. Tissues were placed immediately in RNA-later (Sigma) for gene expression analysis and stored at 4 °C overnight. RNA-later was removed and tissues stored at −70 °C until RNA extraction. A booster vaccination was carried out 315 degree days (dd) post-vaccination (pv) using the same methods as applied during the primary vaccination. Six weeks pv (630 dd), mucus (as above) and blood was sampled from three fish per duplicate group by caudal puncture using a 25G × 16 mm needle (Terumo, Scientific Laboratory Supplies, UK) and 1 mL syringe (Terumo, Scientific Laboratory Supplies, UK). Blood was allowed to clot overnight 4 °C and serum was collected following centrifugation at 3000 × *g* for 5 min and stored at −20 °C until analysed. Tissue samples were collected following bleeding and immediately fixed as above for histology.

### Pre-challenge of unvaccinated fish

The fish were starved for 24 h prior to challenge. Starter cultures were prepared by inoculating MV broth with a cryobead (Protect™, TSC Ltd, UK) of the challenge strain and incubating at 15 °C, 140 rpm for 72 h. This culture was used to inoculate the main culture of MV broth (1:100) which was incubated at 15 °C, 140 rpm for 24 h. Cultures were harvested by centrifugation at 3000 × *g*, 10 min, washed twice with sterile PBS and the OD adjusted to 1 at 525 nm (2 × 10^8^ CFU mL^−1^). One group of 18 unvaccinated fish (7.5 ± 2 g) was immersed in 3 L of tank water containing a solution of hydrogen peroxide at 200 mg L^−1^ for 1 h with aeration. Six of these fish were then placed in a bathing solution of 1 L of *F. psychrophilum* (AVU-1T/07, serotype Th) at 1 × 10^8^, 1 × 10^7^ or 1 × 10^6^ CFU mL^−1^ in static conditions with aeration for 5 h. Fish were then placed in 10 L flow-through tanks for the duration of the challenge (21 days). Moribund fish or mortalities were removed and sampled by streaking head kidney, spleen and any skin lesions on MV agar to confirm specific mortality. A sub-sample of colonies recovered were subjected to 16S rRNA nested PCR for detection of *F. psychrophilum*.

### Immersion challenge and sampling

The fish (12.4 ± 2.3 g) were starved for 24 h prior to challenge (630 dd pv). The bacteria for challenge (recovered from mortalities pre-challenged fish) were cultured as above. Each group of fish (unvaccinated, vaccinated) were immersed in 3 L of tank water containing 200 mg L^−1^ hydrogen peroxide (Sigma) in static aerated 5 L tanks for 1 h. Each group of fish were then immediately placed into a static bath of 5 L of live *F. psychrophilum* (AVU-1T/07) at 1 × 10^8^ CFU mL^−1^ with aeration for 5 h. The fish were then placed back into the holding tanks from which they had originated, were maintained as above and monitored for 32 days. Moribund fish or mortalities were sampled and subjected to bacteriological culture and suspect colonies screened using the PCR assay for *F. psychrophilum* as described above.

### RNA isolation and cDNA synthesis

RNA was extracted from 30 to 40 mg of each tissue sample using TRI Reagent (Sigma, UK) following the manufacturer’s instructions. RNA samples were stored at −70 °C until further use. RNA quantity and quality were determined using the Nanodrop ND-1000 Spectrophotometer (Thermo Fisher Scientific, UK). RNA integrity was checked by gel electrophoresis (1.0% agarose gel containing 0.1 µg mL^−1^ ethidium bromide (Sigma, UK) in 0.5 X Tris–Acetic–EDTA (TAE) buffer [20 mM Tris (Fisher chemicals, UK), 10 mM acetic acid (Fisher chemicals, UK), and 0.5 mM EDTA (Sigma, UK)]. Any potential contaminating DNA in RNA samples were removed using a DNA-free kit (Ambion) according to the manufacturer’s instructions. Synthesis of cDNA was performed using the High Capacity cDNA Reverse Transcription kit (Applied Biosystems, USA) according to the manufacturer’s instructions.

### Quantitative real time PCR (qRT-PCR)

Gill, hind-gut, skin, head-kidney and spleen samples taken 4 h, day 2 and day 7 pv were analysed by qRT-PCR for the expression of immune genes and cell markers (*IgT, IgM, CD4*-*1, CD8α, IL*-*1β, C3, TLR*-*2*). All real-time quantification PCR assays were conducted in white 96-well plates using the Eppendorf^®^ RealPlex2 Mastercycler gradient S instrument with SYBR^®^ Green I master mix (Thermo Scientific, UK) and primers (MWG) as listed in Table 1. A 20-µL reaction mix consisted of 5 µL of cDNA (0.5 ng) and 15 μL of master mix prepared using 1 μL of the forward and reverse primers (0.3 µM each), 10 μL SYBR^®^ Green I and 3 μL of nuclease free water. The cycling conditions consisted of 94 °C for 15 s, followed by 40 amplification cycles of denaturation at 95 °C for 30 s, annealing at the optimal temperature for each primer pair (Table 1) for 30 s and extension at 72 °C for 120 s. Non-template and RT^−^ controls were included on every plate. Melting curve analysis was performed from 60 to 95 °C in 0.1 °C/s increments to assess the specificity of the qRT-PCR products. Serial dilutions of a pool of all cDNA were prepared in nuclease free water starting at 1:5 and the threshold cycle (*Ct*) values were used to generate a standard curve plot of cycle number versus log concentration in the RealPlex software V2.2 (Eppendorf). The quality of the standard curve was judged by the slope of the curve and the correlation coefficient (r). The slope of the line was used to estimate the efficiency of the target amplification using the equation E = (10^−1/slope^) − 1. The expression results were analysed using the 2^−ΔΔ^ Ct method [16]. The gene expression data were normalised to the reference genes (elongation factor-α and β-actin [17]) and expressed as a comparison of vaccinated fish compared to control fish. All the primers used in this study were analysed for self-annealing using NCBI Blast sequence analyser.

**Table 1:** **Details of primers used in RT- qPCR**

Gene	Oligo sequence	Product size (bp)	Annealing temperature (°C)	Genbank accession no.
EF-1α	F: GATCCAGAAGGAGGTCACCA R: TTACGTTCGACCTTCCATCC	150	58	NM_001124339.1
β-actin	F: CAGCCCTCCTTCCTCGGTAT R: AGCACCGTGTTGGCGTACA	110	54	NM_001124235.1
TLR-2	F: GATCCAGAGCAACACTCTCAACAT R: CTCCAGACCATGAAGTTGACAAAC	282	58	LK933545.1
C3	F: GAGATGGCCTCCAAGAAGATAGAA R: ACCGCATGTACGCATCATCA	91	58	L24433.1
IL1β	F: GACATGGTGCGTTTCCTTTT R: ACCGGTTTGGTGTAGTCCTG	122	54	AJ278242.2
IgT	F: AACATCACCTGGCACATCAA R: TTCAGGTTGCCCTTTGATTC	80	54	AY870266.1
IgM	F: TGCGTGTTTGAGAACAAAGC R: GACGGCTCGATGATCGTAAT	107	54	AH014877.2
CD4-1	F: GAGTACACCTGCGCTGTGGAAT R: GGTTGACCTCCTGACCTACAAAGG	121	58	NM_001124539.1
CD8a	F: ACGACTACACCAATGACCACAACC R: CAGTGATGATGAGGAGGAGGAAGA	160	58	AF178055.1

### Immunohistochemistry for the detection of *F. psychrophilum* and IgT positive cells

Tissue sections were dewaxed and dehydrated and incubated with 3% hydrogen peroxide (H_2_O_2_) in methanol for 10 min at 22 °C to block endogenous peroxidases. Slides were washed three times with Tris Buffered Saline (TBS:10 mM Tris base, 0.5 M NaCl, pH 7.5) and non-specific binding sites blocked with 2% BSA (Bovine Serum Albumin) in TBS for 10 min followed by normal goat serum diluted 1:10 in TBS for 10 min at room temperature. Detection of *F. psychrophilum* antigen: 50–100 μL of anti-*F. psychrophilum* polyclonal antibody (1:200) was placed onto the tissue sections and incubated for 1 h at room temperature in a humid chamber. Slides were washed as above and incubated with streptavidin–horseradish peroxidase (1:200) for 1 h. Slides were washed as described above. To visualise the reaction, slides were incubated for 10 min with DAB solution followed by rehydration and mounting. For the detection of IgT, 50–100 μL of mouse anti-IgT monoclonal antibody (ADL Ltd.) was applied to tissue sections according to [18].

### Detection of specific IgM in serum (ELISA)

To assess specific IgM titers in serum at 630 dd, an enzyme-linked immunosorbent assay **(**ELISA) was performed according to [19].

### Detection of total IgT in serum and skin mucus (ELISA) and specific IgT in skin mucus (Western blot)

To assess the total IgT levels in serum at 630 dd an ELISA was performed according to [20]. Serum samples from duplicate tanks (n = 6) were pooled for analysis. To assess specific IgT in skin mucus a Western blot was performed according to [20]. Mucus from unvaccinated and vaccinated fish 630 dd was incubated overnight at 4 °C with the strains of *F. psychrophilum* used in the vaccine (neat, 1:1 and 1:10) in a pull-down assay before being run on an SDS-PAGE gel.

### Statistical analysis

SPSS and Microsoft Excel were used to generate graphs and for statistical analysis. The relative standard deviation (RSD = (standard deviation/mean) × 100) was calculated for mortality rates of two groups after the bath challenge. Kaplan–Meier survival curves were generated and the log-rank test was used to compare the survival curves for the vaccinated fish and unvaccinated fish [21, 22]. The relative percent survival (RPS) of this trial was calculated using the following equation [23]:

$$ {\text{RPS}} = \left[ {1 - \frac{{{\text{average }}\% {\text{ mortality of vaccinated fish}} }}{{{\text{average }}\% {\text{ mortality of unvaccinated fish }}}}} \right] \, \times { 1}00 $$

The Fischer’s exact test was carried out on survival data between individual tanks. Normality of serum and mucus antibody responses was checked using the Kolmogorov–Smirnov test and differences between groups was analysed by the Student’s two-tailed unpaired *t* test. To determine the limit of quantification (LOQ) in the ELISA for IgM and IgT, 6 standard deviations (S.D.) were added to the absorbance readings of the blank wells.

## Results

### Characterisation and selection of *F. psychrophilum* isolates for inclusion in the vaccine

A high strain diversity was identified among the isolates with 54 pulsotypes, ten (GTG) 5-PCR types, two 16S rRNA allele lineages, seven plasmid profiles and three serotypes [8]. The predominant profile observed within the *F. psychrophilum* isolates examined was PFGE cluster II_(GTG)5-PCR type r1—16S rRNA lineage II_serotype Th (70/156 isolates examined, 45%). Co-existence of genetically and serologically heterogeneous isolates within each farm was detected. Therefore to cover the diversity observed within the isolates we selected two trout and one salmon isolate, three serotypes Th, Fp^T^, Fd; and three different pulsotypes for inclusion in the vaccine.

### Vaccine efficacy

**Figure 1:**
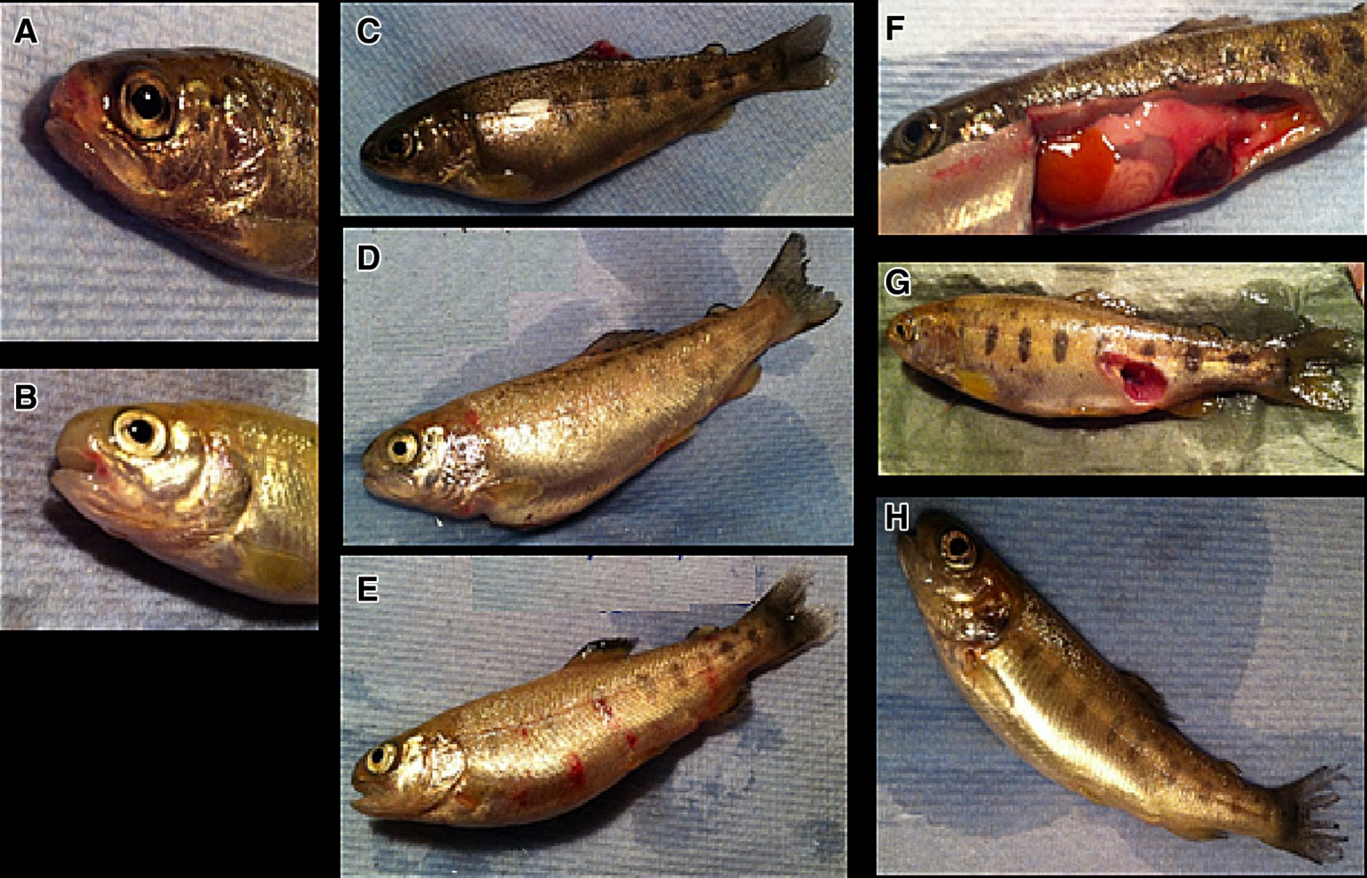
**Clinical signs of RTFS following bath challenge with a heterologous** ***F. psychrophilum*** **strain AVU-1T/07.** Eroded mouth (**A**); hemorrhage on mouth (**B**), dorsal fin (**C**), operculum, pelvic fin (**D**) and the trunk (**E**); splenomegaly and blurred spleen margins (**F**); lesion on the trunk (**G**); eroded tail (**H**).

**Figure 2:**
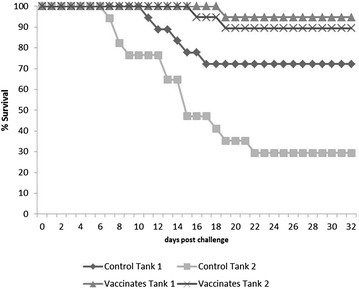
**Vaccine efficacy.** Percentage survival after bath challenge of replicate vaccinated and control groups with *F. psychrophilum* strain AVU-1T/07. The survival rates from each replicate tank following challenge are presented, p = 0.0002; RPS 84%.

The fry were found to be negative for the presence of *F. psychrophilum* by nested PCR. Fish were challenged with *F. psychrophilum* isolate AVU-1T/07 (1 × 10^8^ CFU mL^−1^) 630 degree days post primary vaccination. Mortalities started 8 days post-challenge (pc) in the control groups and continued until day 23. In contrast, mortalities in the vaccinated groups occurred 17 and 20 days pc. All the moribund and dead fish showed at least one of the following clinical signs: haemorrhage, splenomegaly, lesion on the trunk, and/or eroded tail and mouth (Figure 1). The presence of *F. psychrophilum* was detected by bacteriological culture and 16S rRNA nested PCR in all moribund and dead fish sampled during the challenge. No colonies recovered from any of 16 representative survivors (four fish per replicate group) following the termination of the challenge were positive for *F. psychrophilum* by PCR. The average cumulative mortality was 49% in unvaccinated group and 8% in the vaccinated group (Tables 2 and 3). The survival rates from each tank following challenge are presented as a Kaplan–Meier plot (Figure 2), *p* = 0.0002. The calculated RPS induced by the polyvalent inactivated vaccine was 84%.

**Table 2:** **Average percent mortality of rainbow trout fry after bath challenge with a heterologous** ***F. psychrophilum*** **strain AVU-1T/07**

Group	Replicate tank	Weight (g)	*n*	% mortality (n)	Average % mortality	RSD (%)
Control	1	12.46 (±3.25)	18	27.78 (5)	49.185 (±30.27)	61.54
	2	12.75 (±2.43)	17	70.59 (12)		
Vaccinated	1	12.08 (±2.02)	19	5.26 (1)	7.895 (±3.73)	47.25
	2	12.47 (±1.49)	19	10.53 (2)		

Average values for weight and mortality rate are stated along with standard deviation. *n* represents the number of fish.

*RSD* relative standard deviation.

**Table 3:** **Fischers exact test showing comparison of survival between individual tanks**

Fischers exact test	Vaccinate tank 1	Vaccinate tank 2
Control Tank 1	0.09	0.232
Control Tank 2	0.000	0.000

### Gene expression

The relative expression of *IgT*, *IgM*, *CD4*-*1*, *CD8α*, *IL*-*1β*, *C3*, *TLR*-*2* was normalised against *EF*-*1α* and *β*-*actin*. The relative fold change in gene expression of these genes in gill, hind-gut, skin, head kidney and spleen at early time points pv is summarised in Table 4. At 4 h pv a significant up-regulation of *IL*-*1β* was detected in head-kidney (*p* < 0.05). Expression of *IgT* and *IgM* was significantly up-regulated in hind-gut at day 2 in conjunction with a significant up-regulation of *TLR*-*2* expression in the skin of immersion vaccinated fish when compared to unvaccinated fish (*p* < 0.05). *IL*-*1β* was down-regulated in gill day 2 pv (*p* < 0.05), *CD8α* significantly down-regulated at day 2 in head-kidney (*p* < 0.05), and *CD4*-*1* (*p* < 0.01) and *CD8α* (*p* < 0.05) expression significantly down-regulated at day 7 in the spleen.

**Table 4:** **Gene expression in five organs of rainbow trout fry at 4** **h, day 2 and day 7 post-initial vaccination**

	4 h	Day 2	Day 7
Gill			
TLR-2	1.835 ± 0.48	2.427 ± 0.73	1.776 ± 0.73
C3	0.878 ± 0.61	1.06 ± 0.76	1.215 ± 0.66
IL-1β	0.654 ± 0.41	*0.512* ↓ ± 0.34	0.651 ± 0.43
IgT	1.041 ± 0.64	1.096 ± 0.72	0.713 ± 0.50
IgM	0.713 ± 0.34	1.344 ± 0.85	1.088 ± 0.66
CD4	0.914 ± 0.66	0.975 ± 0.83	0.922 ± 0.70
CD8	0.81 ± 0.58	0.76 ± 0.38	1.071 ± 0.58
Hind-gut			
TLR-2	1.808 ± 0.55	1.5 ± 0.86	0.562 ± 0.30
C3	0.444 ± 0.09	0.485 ± 0.163	0.611 ± 0.15
IL-1β	1 ± 0.51	1.018 ± 0.54	0.678 ± 0.37
IgT	0.742 ± 0.32	*2.050* ↑ ± 1.23	0.23 ± 0.02
IgM	1.381 ± 0.72	*2.035* ↑ ± 1.16	0.51 ± 0.15
CD4	1.126 ± 0.93	1.476 ± 1.01	0.853 ± 0.46
CD8	0.873 ± 0.54	0.748 ± 0.37	0.538 ± 0.25
Skin			
TLR-2	2.08 ± 0.89	*4.348* ↑ ± 1.82	1.611 ± 0.53
C3	1.038 ± 0.73	0.958 ± 0.83	1.093 ± 0.85
IL-1β	1.019 ± 0.59	0.475 ± 0.23	0.971 ± 0.54
IgT	1.183 ± 0.75	1.322 ± 0.54	0.491 ± 0.24
IgM	0.73 ± 0.53	1.729 ± 0.77	1.1 ± 0.34
CD4	0.772 ± 0.46	1.272 ± 0.88	0.974 ± 0.71
CD8	0.679 ± 0.34	0.711 ± 0.31	0.713 ± 0.42
Head kidney			
TLR-2	1.031 ± 0.45	0.918 ± 0.56	0.791 ± 0.50
C3	0.677 ± 0.10	1.444 ± 0.43	5.26 ± 0.67
IL-1β	*1.819* ↑ ± 0.90	1.001 ± 0.42	1.347 ± 0.64
IgT	0.995 ± 0.51	1.199 ± 0.85	2.189 ± 0.20
IgM	0.988 ± 0.71	0.784 ± 0.53	0.654 ± 0.42
CD4	0.944 ± 0.66	1.128 ± 0.83	1.191 ± 0.83
CD8	0.788 ± 0.56	*0.426* ↓ ± 0.21	0.947 ± 0.47
Spleen			
TLR-2	1.823 ± 0.54	2.017 ± 0.687	1.037 ± 0.37
C3	0.793 ± 0.39	1.205 ± 0.63	0.67 ± 0.32
IL-1β	2.494 ± 0.98	0.734 ± 0.44	1.08 ± 0.75
IgT	0.898 ± 0.41	1.572 ± 0.737	1.23 ± 0.46
IgM	1.184 ± 0.60	0.734 ± 0.34	0.649 ± 0.41
CD4	0.869 ± 0.63	0.935 ± 0.61	*0.782* ↓↓ ± 0.61
CD8	0.701 ± 0.46	0.469 ± 0.24	*0.554* ↓ ± 0.36

Fold change of genes in vaccinated groups compared to controls ± SE.

↑ and ↓ indicate significant up-regulation and down-regulation relative to control respectively (p < 0.05). ↓↓ indicate significant up-regulation and down-regulation relative to control respectively (p < 0.01).

### Immunohistochemistry

#### Detection of *F. psychrophilum* in mucosal tissues of vaccinated fish

**Figure 3:**
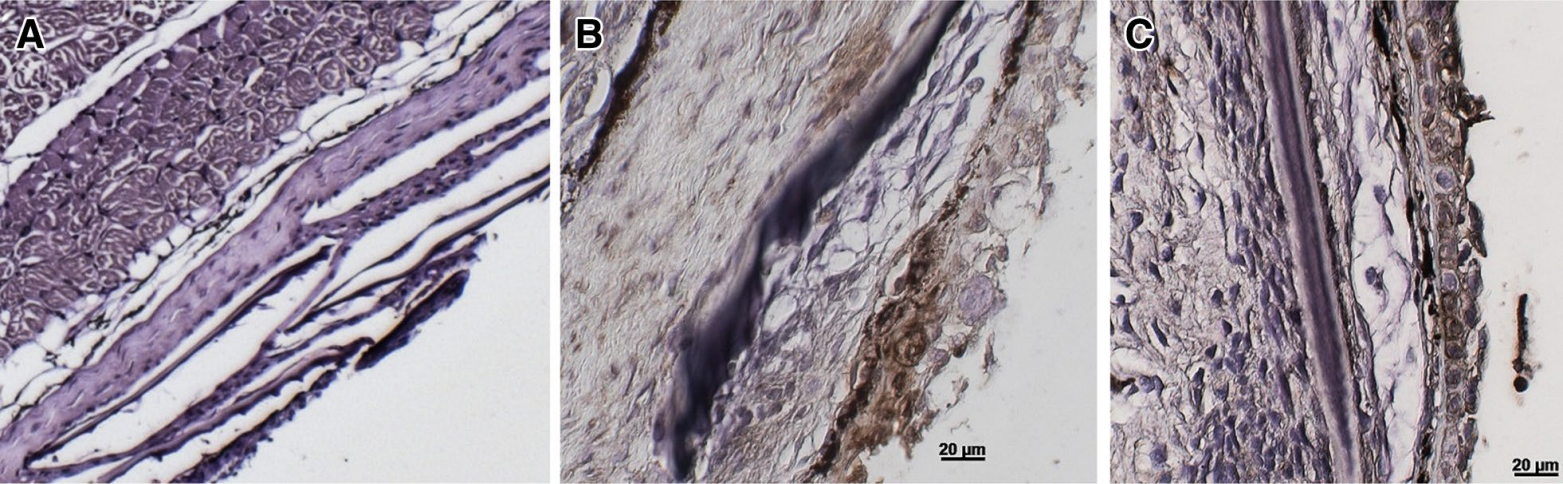
**Immunohistochemical detection of antigen uptake following immersion vaccination in skin of trout.** Detection of *F. psychrophilum* antigen in skin of trout at **A** unvaccinated **B** 4 h pv and **C** day 2 pv. Brown staining indicates positive staining for antigen. Bar: 20 µm.

**Figure 4:**
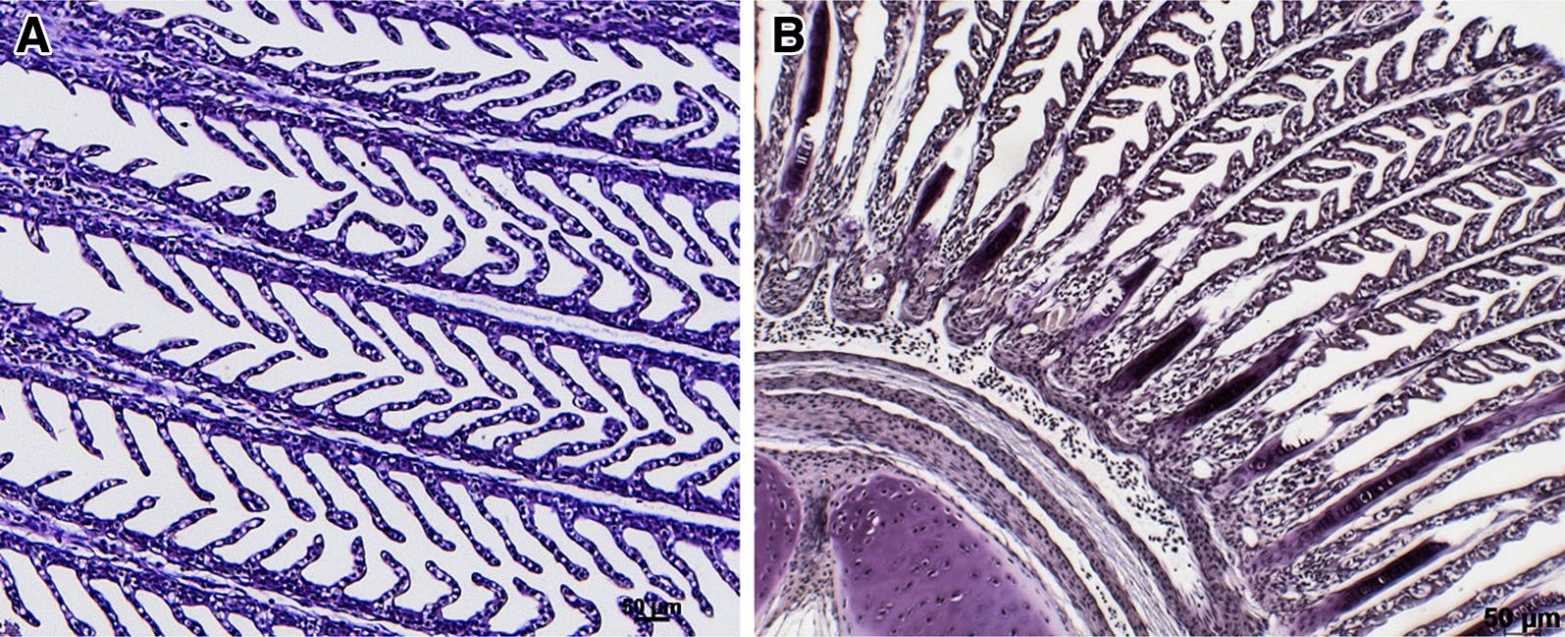
**Immunohistochemical detection of antigen uptake following immersion vaccination in gill of trout.** Detection of *F. psychrophilum* antigen in gills: **A** unvaccinated, **B** day 2 pv. Brown staining indicates positive staining for antigen. Bar: 50 µm.

**Figure 5:**
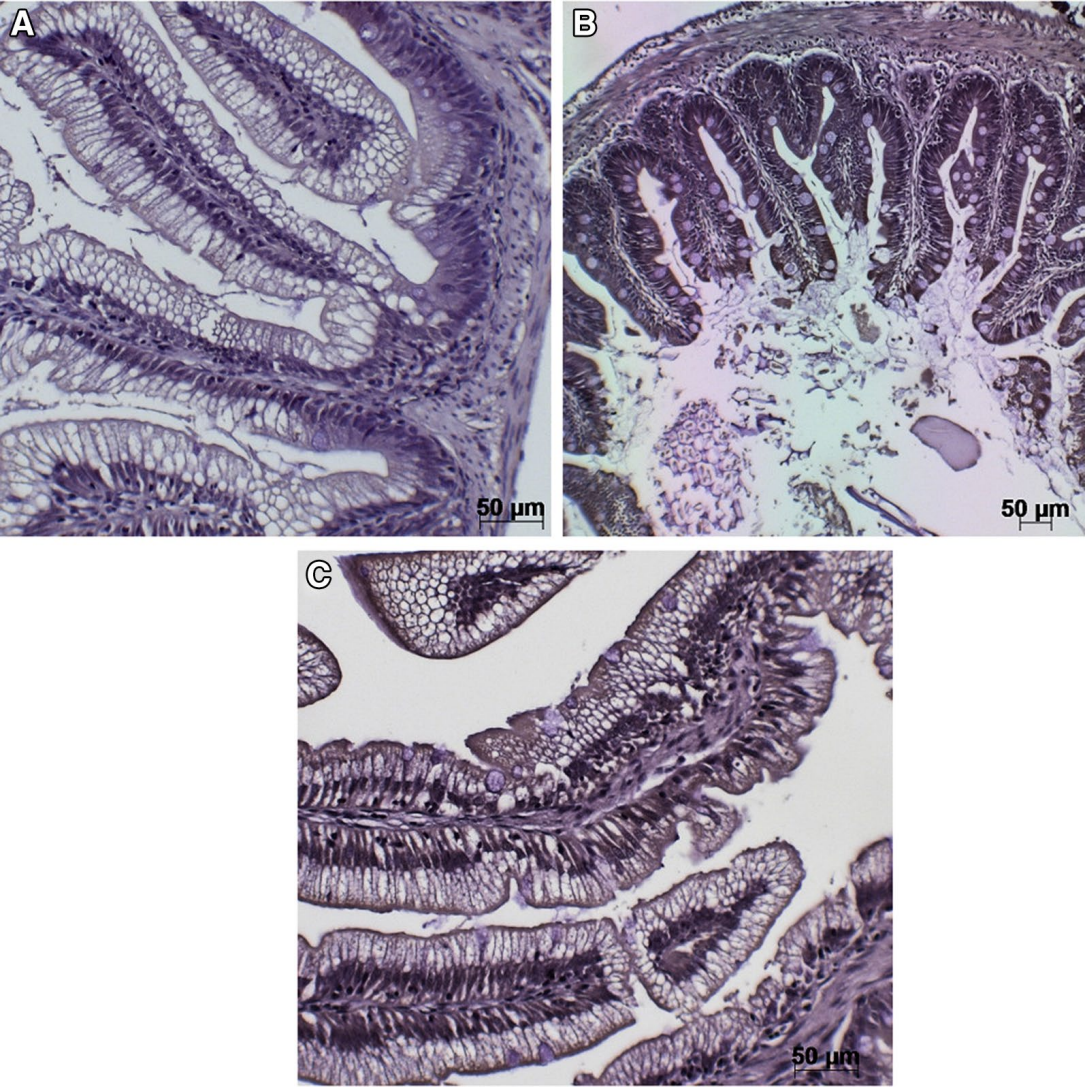
**Immunohistochemical detection of antigen uptake following immersion vaccination in hind-gut of trout.** Detection of *F. psychrophilum* antigen in hind-gut: **A** unvaccinated **B** day 2 pv and **C** day 7 pv. Brown staining indicates positive staining for antigen. Bar: 50 µm.

Skin (1.5 cm^3^ section just to the left of and below the dorsal fin), gill and hind-gut samples were examined for the presence of *F. psychrophilum* at 4 h, day 2 and day 7 pv by immunohistochemistry (IHC). Staining by IHC for *F. psychrophilum* was observed in the skin epithelium at 4 h and day 2 pv (Figures 3B and C). Positive staining for *F. psychrophilum* was observed at day 2 pv on the gills (Figure 4B) and at day 7 no staining was observed. Positive staining for *F. psychrophilum* was seen in the hind-gut (enterocytes) by day 2 pv and increased in strength by day 7 pv (Figures 5B and C).

#### Detection of IgT positive cells in mucosal tissues of vaccinated fish

**Figure 6:**
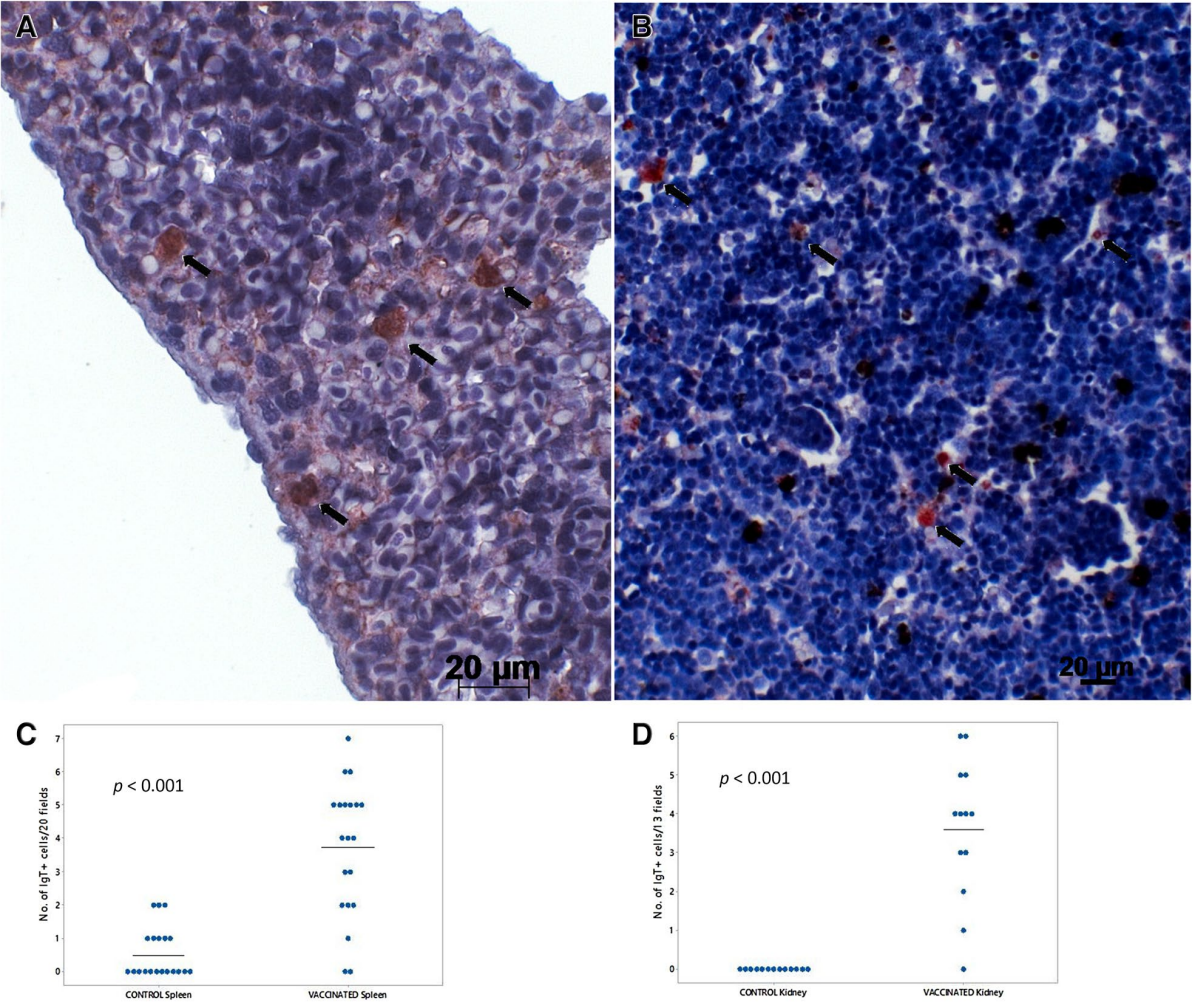
**Immunohistochemical detection of trout IgT** ^**+**^ **cells in systemic organs following immersion vaccination.** Detection of IgT^+^ cells in **A** spleen and **B** head-kidney of vaccinated trout 6 wpv are depicted. Graphs show numbers of IgT^+^ cells in **C** spleen and **D** head-kidney 6 wpv (n = 3). Number of cells in vaccinated fish significantly higher than the number in corresponding controls (*p* < 0.001), line indicates average number cells. Arrows indicate positive staining. Bar: 20 µm.

Skin, gill, hind-gut, spleen and head-kidney samples were examined for the presence of IgT^+^ cells at day 7 and 6 weeks pv by immunohistochemistry. Numbers of IgT^+^ cells detected in spleen and head-kidney of immersion vaccinated fish were found to be significantly higher than unvaccinated fish at 6 wpv (*p* < 0.001) (Figures 6A–D). IgT^+^ cells were not observed in the skin of vaccinated or unvaccinated fish. Occasional IgT^+^ cells were observed in the gill and hind-gut of fish however there was no significant difference between treatment groups.

#### ELISA for detection of specific IgM in serum

**Figure 7:**
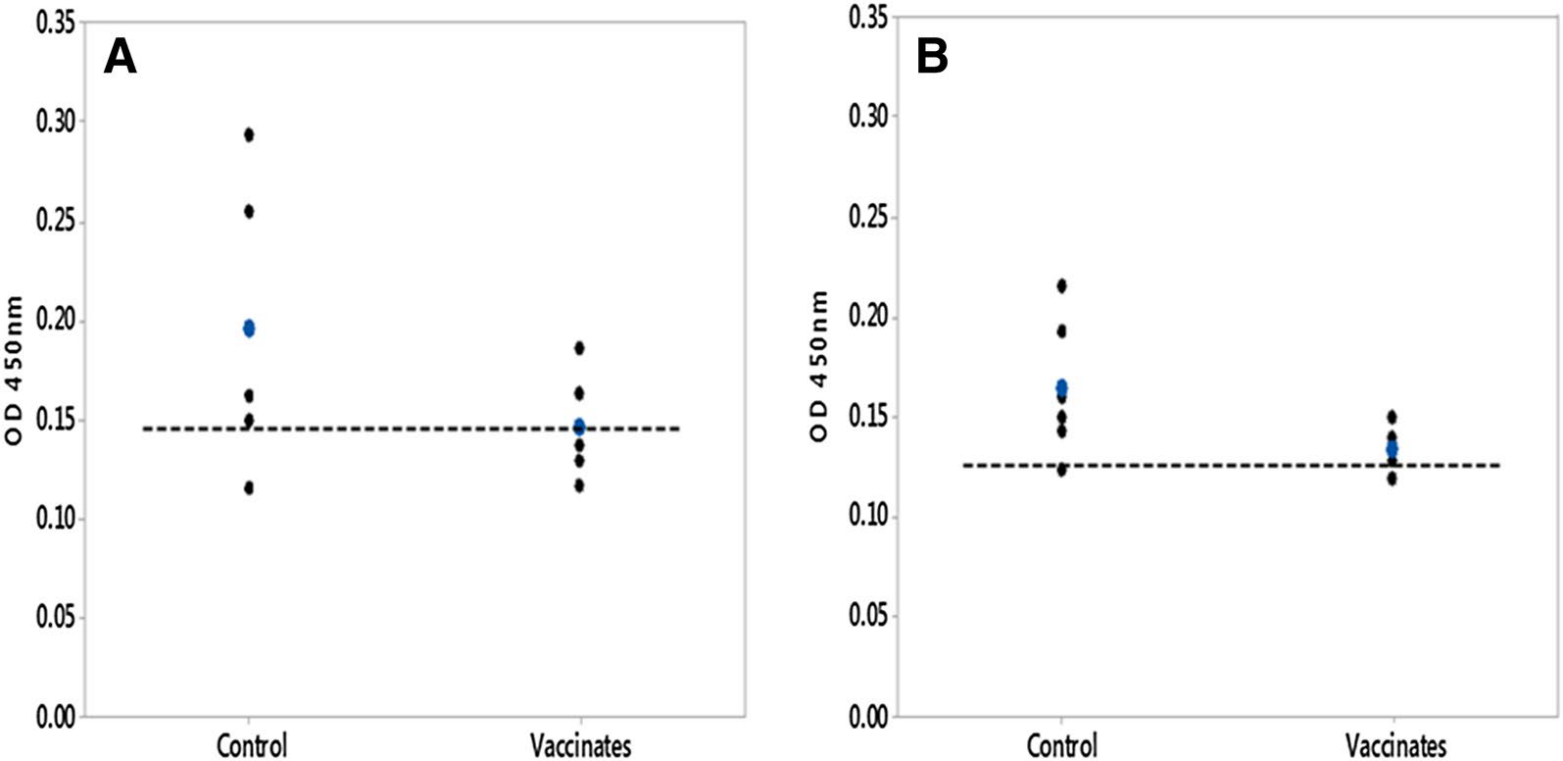
**Specific IgM serum responses of trout fry following immersion vaccination. A** Serum (6 weeks pv) IgM response to vaccine strain AVU-1T/13; **B** to vaccine strain AVU-2T/13. Serum dilution was 1:32. Dashed line represents the LOQ, blue dots are mean values.

Specific IgM levels in serum 6 weeks pv to both trout *F. psychrophilum* strains in the vaccine were not significantly different in the vaccinated group when compared to the unvaccinated group (Figure 7).

#### Detection of total IgT in serum (ELISA) and specific IgT in skin mucus (Western blot)

**Figure 8:**
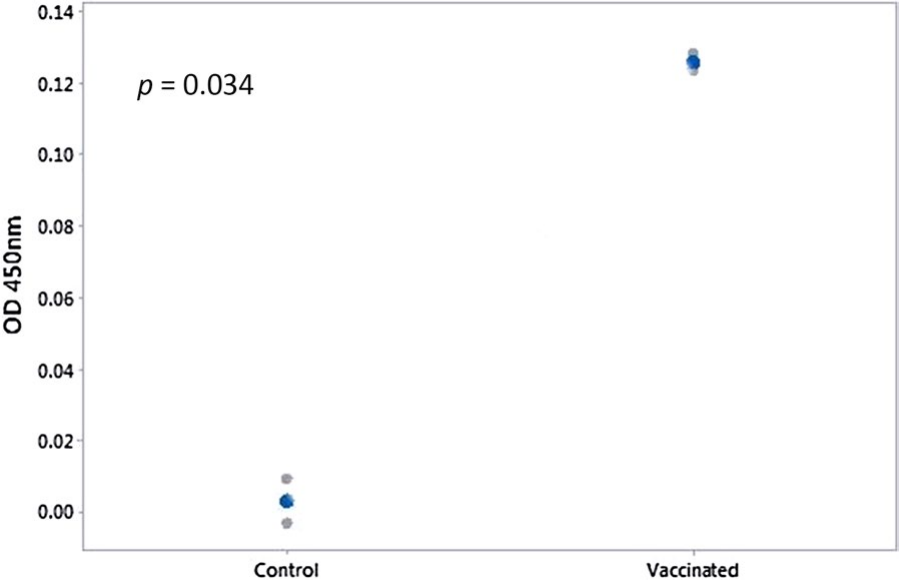
**Total IgT in serum of immersion vaccinated fish compared to unvaccinated fish by ELISA.** Serum (6 weeks pv) from fish from duplicate tanks were pooled for analysis (n = 2) and diluted 1:20. Total serum IgT in vaccinated fish was significantly higher than in controls (*p* = 0.034), blue dots are mean values.

Levels of total serum IgT were low but significantly higher in immersion vaccinated fish serum when compared with unvaccinated fish 6 wpv (*p* = 0.034) (Figure 8). Specific IgT was not detectable in skin mucus sampled 6 wpv from vaccinated or unvaccinated fish by Western blot (Additional file 1).

## Discussion

Rainbow trout fry syndrome caused by *F. psychrophilum* is widespread in both rainbow trout and Atlantic salmon during the early stages of production when the fish are too small to vaccinate by injection. In addition, the high diversity of this pathogen present in the UK and elsewhere as evidenced by [8] further complicate the development of a vaccine. Ideally, an immersion or oral vaccine to protect the fish at the fry stage is urgently needed. Developing highly protective mucosal vaccines is more challenging than injectable vaccines due to dose considerations, application methods and difficulties in collecting and measuring the immune response in mucus. Ideally vaccines delivered by mucosal routes would have the capacity to stimulate both mucosal and systemic immunity thereby protecting the fish at the portal of entry of pathogens and preventing the spread of infection systemically [24].

We describe a new polyvalent whole cell vaccine developed by combining three genetically and serologically different *F. psychrophilum* strains. The polyvalent vaccine was shown to provide significant protection (RPS 84%) against a heterologous *F. psychrophilum* strain when administered to rainbow trout fry (mean weight of 4.7 ± 1.0 g) by immersion. Furthermore, mortality in vaccinated fish was delayed by 9–12 days compared with unvaccinated fish. The RPS value (84%) obtained in this study was higher than those of previous studies using a similar administration route (14–47% [25]; 13% [26]; 28–45% [27]). Another study [28] observed a considerably high RPS value (88%) resulting from the immersion exposure of fry in a combination of two live *F. psychrophilum* isolates, while no protection was seen in the group immersed in the inactivated vaccine. The low RPS values obtained in studies involving an immersion vaccination may be due to the incompatible challenge procedures used to determine efficacy, such as intraperitoneal or subcutaneous injection, which bypass the mucosal immunity stimulated by immersion vaccination. In addition, a prime-boost vaccination strategy was applied in this study as previous successful immersion vaccines developed for fish have demonstrated the need for boosting following immersion [29–31]. This is probably due to the lower dose of antigen delivered by the immersion route as opposed to injectable vaccines and the lack of adjuvant to induce an acute inflammatory response. In addition, in-depth characterisation to enable selection of vaccine candidates for such a heterogeneous pathogen is crucial. The previous study [8] carried out prior to selection of isolates to be included in the vaccine characterised a large collection of isolates (315) by several methods (genotyping and serology). This allowed the “big picture” of what was happening in the field with *F. psychrophilum*, especially in the UK, to be examined and the isolates were chosen on the basis of which would best represent the heterogeneity observed within those found circulating in the field.

In order to evaluate the efficacy of an immersion vaccine for RTFS a reproducible experimental bath infection for *F. psychrophilum* is essential. Previous attempts to standardise a bath challenge model for *F. psychrophilum* with consistently high mortalities have met with limited success unless scarification or stress has been used [9, 10, 12, 32]. A study in 2013 using hydrogen peroxide as a pre-stressor resulted in a twofold increase in mortality up to 30% [14]; however this is insufficient to test the efficacy of a vaccine [23]. In addition, the fish used in the 2013 study were between 0.6 and 1.5 g whereas the size of fish following a vaccination trial will inevitably be larger. In the present study, we used hydrogen peroxide as a pre-stressor but modified the time of immersion exposure to *F. psychrophilum* from 30 min to 5 h using 5–6 g trout in preliminary trials. This method consistently produced mortalities of between 50 and 60% with different isolates of *F. psychrophilum*; clinical signs of the disease were evident and *F. psychrophilum* was reliably recovered from the challenged fish. Therefore, we used this model to test the efficacy of our immersion vaccine and the average mortality was nearly 50% in the control groups. However, the relative standard deviation (RSD) in mortality between replicate tanks was high in both unvaccinated (61.5%) and vaccinated fish (47.25%). One factor which may have affected the reproducibility of the challenge was the size of the fish. Due to unforeseen circumstances, the fish were nearly 5 g when the vaccination trial began, resulting in the average weight of the fish at time of challenge being 12 g, which is twice the weight at which the challenge dose had been determined. Further work is clearly warranted in standardising the model for use with larger fish including more replicates and larger numbers of animals.

The route of entry of live *F. psychrophilum* seems to be dependent on a breach of the epithelium (either skin or gill) to enter the host. A study which looked at experimentally infected fins of Atlantic salmon revealed *F. psychrophilum* bacteria were embedded in the mucus layer where this was intact on the skin surface; whereas invading bacteria were seen burrowing into the fin rays where the mucus layer was absent [33]. Indeed pre-treatment with hydrogen peroxide appears to influence the immune response and postpone healing of gill tissue in trout [34] which may have aided uptake of bacteria during challenge.

In an attempt to determine how the novel vaccine developed here protected the fish from challenge with *F. psychrophilum*, we undertook a number of investigations the first being to determine the route of antigen uptake in the fish pv. Mucosal tissues were sampled and subjected to immunohistochemistry to detect the vaccine antigen. Positive staining for *F. psychrophilum* was evident coating the gill lamellae day 2 pv suggesting that the mucus surrounding the gills may have trapped the antigen thereby retaining the bacteria. Bacterial antigen staining in the skin of vaccinated fish was evident 4 h and day 2 pv. Uptake of antigen through the hind-gut was also evident by staining of enterocytes which was observed at day 2 pv and increased in intensity by day 7. Previous studies on uptake of *Y. ruckeri* demonstrated tissue specific uptake by IHC and in situ hybridisation following exposure to formalin killed and live bacteria [35].

Other studies have shown that the gut of teleosts has a developed if diffuse repertoire of immune cells including APCs such as monocytes, macrophages in the lamina propria (LP) and intra-epithelial lymphocytes (IELs) [36]. Immersion vaccination of zebrafish, *Danio rerio*, with live attenuated *V. anguillarum* revealed that bacteria persisted in the gut while levels of bacteria decreased rapidly in skin and gill [37]. APCs in the gut of the zebrafish also displayed active responses in antigen recognition and sampling following vaccination. In addition, it is important to note that the vaccination in the current study was performed at 15 °C, which is in the higher temperature range of rainbow trout which may have allowed increased vaccine uptake. This effect was seen in a study of immersion vaccination of rainbow trout against *V. anguillarum* which found that the uptake of vaccine is correlated with temperature; uptake was significantly lower at low temperature (5 °C) than uptake at 18 °C [38]. Temperature at vaccination will also influence the secondary immune response and it has been shown that fish can produce a secondary antibody response at low temperatures if they had previously encountered the antigen at high temperatures [39–42].

The importance of the innate response in activating specific immunity is now understood [43] and therefore the activation and re-distribution of phagocytes is highly significant especially in the context of vaccination. The rapid activation of pro-inflammatory factors in the present study was evidenced by the early (4 hpv) up-regulation of the cytokine *IL*-*1β* in the head-kidney which is produced by activated macrophages [44]. Furthermore up-regulation of Toll-like receptor 2 in the skin of vaccinated fish was observed. TLRs are receptors which recognise pathogen associated molecular patterns (PAMPS) and thereby trigger signalling pathways which activate immune cells in response to infection. TLR-2 is involved in detecting highly conserved structures of bacterial origin [45]. In the present work, the amount of *TLR*-*2* transcripts in skin was significantly up-regulated 2 days pv, indicating that inactivated *F. psychrophilum* cells, or parts thereof, present in the developed vaccine were recognised by TLR-2 receptors on innate immune cells in the skin.

Specific IgM levels in serum of immersion vaccinated fish did not differ significantly from unvaccinated controls at 6 wpv as reported previously for mucosal vaccination [46, 47]. Similar results were obtained in another study of immersion vaccinated trout where only intraperitoneal vaccination resulted in elevated titres of IgM in the serum even with live attenuated *F. psychrophilum* [6]. In contrast, protection against *Y. ruckeri* in immersion vaccinated trout was shown to be strongly associated with plasma IgM levels suggesting the stimulation of IgM responses by the immersion route in trout could be pathogen specific [48].

Down regulation of CD8α and CD4-1was observed in lymphoid organs 2–7 days pv. The effect of the repression of CD8α and CD4-1 following an immersion vaccination using formalin-killed *F. psychrophilum* cells is unclear but could be related to the low titre of IgM in serum, as suggested by previous authors [49].

It is possible that the high RPS seen in the present study could be partially due to the contribution of non-specific immune mechanisms and the induction of the mucosal antibody IgT, as evidenced by proliferation of IgT positive cells in systemic organs, up-regulation of IgT in the hind-gut and an increase of total IgT in the serum of vaccinated fish. IgT is thought to be a specialised immunoglobulin in mucosal immunity in rainbow trout, equivalent to IgA in mammals [20]. A previous study revealed that IgT^+^ B cells were prevalent in the gut of rainbow trout and represented 54% of all B cells [20]. The up-regulation of IgT in hind-gut day 2 pv in the current study suggests that IgT might contribute to the immediate protection of the host at mucosal surfaces against the natural infection route of pathogens. Additionally, the significant up-regulation of IgT as early as 2 days pv, supports the hypothesis that IgT producing B lymphocytes are present locally, thus resulting in increased expression of this immunoglobulin upon stimulation by bath vaccination [6]. While a significant increase in total serum IgT was observed, it must be noted that specific IgT was undetectable in the skin mucus by western blot in the present study. Therefore a functional role of IgT in the observed protection in the present study has yet to be demonstrated. More sensitive techniques to measure IgT levels in serum and mucus are needed.

Addition of an adjuvant may have increased the antigen uptake and processing and hence antibody response to vaccination as seen in immersion vaccination of trout against *V. anguillarum* [38] and *Y. ruckeri* using an immersion adjuvant (Seppic) [50]. Clearly further studies on this and other potential mucosal adjuvants for fish are warranted.

This preliminary study is one of the first to demonstrate the efficacy of a polyvalent inactivated whole cell vaccine against a heterologous strain of *F. psychrophilum* for rainbow trout fry following immersion immunisation and immersion challenge (RPS of 84%). Priming of the non-specific immune response was indicated by a significant up-regulation of *IL1*-*β* in head-kidney and *TLR*-*2* in skin of vaccinated fish. The immersion vaccine induced a significant increase in IgT expression in hind-gut, proliferation of IgT positive cells in systemic organs and an increase of total IgT in the serum of vaccinated fish. Further challenge studies are needed with other serotypes/genotypes of *F. psychrophilum* to estimate the potential of the vaccine to cross protect and to determine the role of IgT in vaccine induced protection for RTFS. A trial is underway to test the efficacy of the vaccine in fry (1.5–2 g) with larger numbers of animals and to standardise the immersion challenge model for *F. psychrophilum.*

## Additional file

**Additional file 1.** Specific IgT (against *F. psychrophilum* antigen) was not detectable in skin mucus (neat, 1:1, 1:10) sampled from unvaccinated or vaccinated fish by Western blot. (A) blot incubated with TBS, (B) blot incubated with mucus.
