# Supplementary figures and images for: Efficacy of a polyvalent immersion vaccine against Flavobacterium psychrophilum and evaluation of immune response to vaccination in rainbow trout fry (Onchorynchus mykiss L.)
Source: Vet Res. 2017 Aug 18;48:43. doi: 10.1186/s13567-017-0448-z (PMC5563058; doi:10.1186/s13567-017-0448-z)

## Slide 1
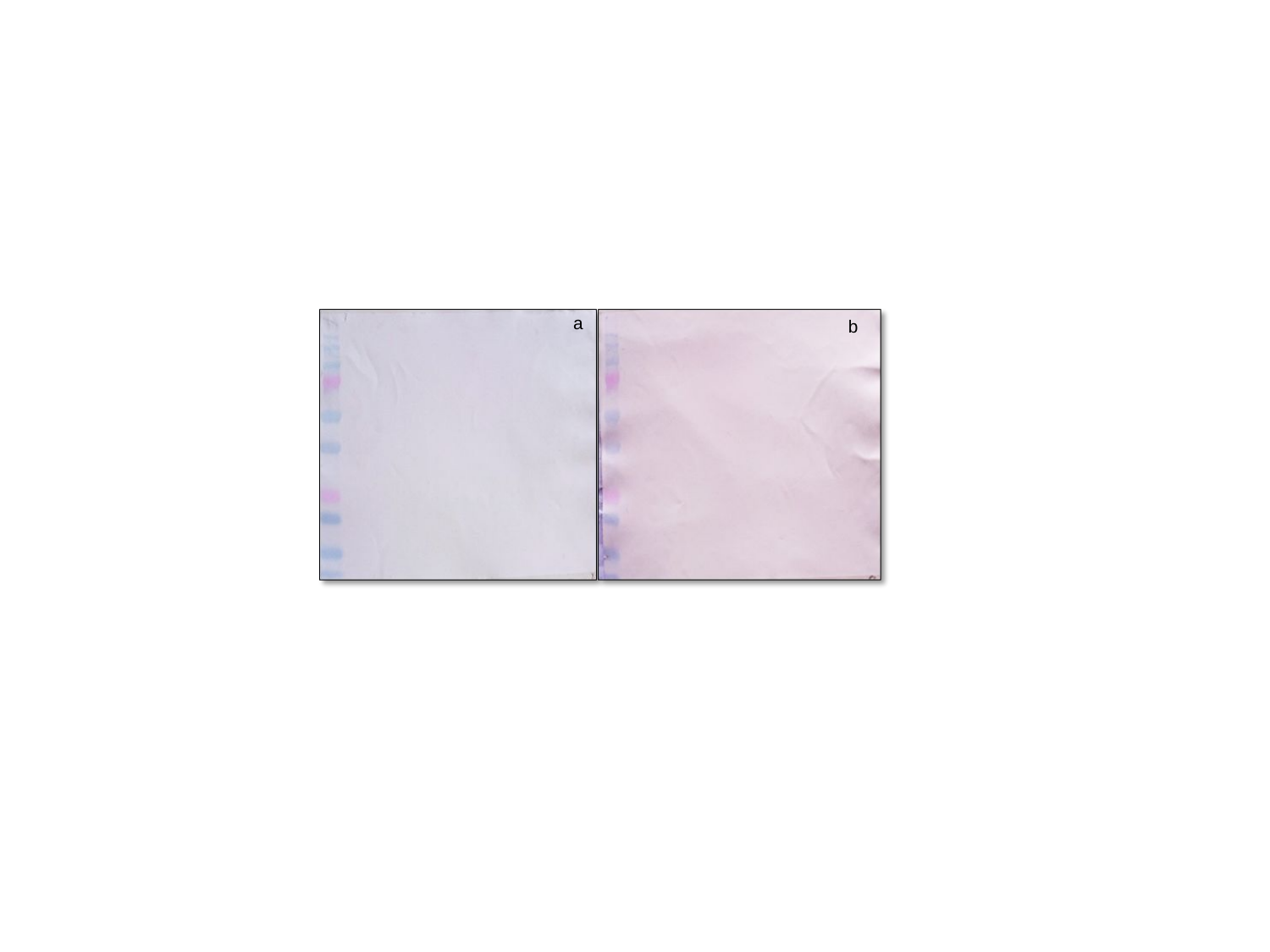

a
b

Supplement: Supplementary file 1 — Additional file 1. Specific IgT (against F. psychrophilum antigen) was not detectable in skin mucus (neat, 1:1, 1:10) sampled from unvaccinated or vaccinated fish by Western blot. (A) blot incubated with TBS, (B) blot incubated with mucus. [file 13567_2017_448_MOESM1_ESM.pptx]
